# Teprotumumab-trbw as a Novel Monoclonal Antibody for Thyroid Eye Disease: A Literature Review

**DOI:** 10.7759/cureus.43878

**Published:** 2023-08-21

**Authors:** Brooke E Bocklud, Waddih Fakhre, Brennen Murphy, Kirsten Maddox, Shahab Ahmadzadeh, Omar Viswanath, Giustino Varrassi, Sahar Shekoohi, Alan D Kaye

**Affiliations:** 1 School of Medicine, Louisiana State University Health Sciences Center, Shreveport, USA; 2 Anesthesiology, Louisiana State University Health Sciences Center, Shreveport, USA; 3 Pain Management, Valley Pain Consultants - Envision Physician Services, Phoenix, USA; 4 Pain Medicine, Paolo Procacci Foundation, Rome, ITA

**Keywords:** grave’s disease, proptosis, teprotumumab, tepezza, thyroid eye disease

## Abstract

Thyroid eye disease (TED) can cause disfigurement and vision loss, most commonly in patients with Graves’ disease. These symptoms are related to orbital inflammation subsequently cause proptosis and limited eye movement. Traditionally, TED is treated with corticosteroids to decrease inflammation and surgery once the disease stabilizes. However, multiple medications that play a role in immune modulation have been tested and found to be beneficial in treating TED, either as an adjuvant to steroids or in severe disease resistant to steroids. Teprotumumab-trbw, a novel monoclonal antibody sold under the trade name Tepezza^®^, is the first immune modulator to be approved by the Unites States Food and Drug Administration (FDA) for TED. Teprotumumab-trbw targets the insulin-like growth factor-1 receptor, which is upregulated on orbital fibroblasts and decreases activation in patients with TED. The FDA approved this drug for patients with less than nine months of disease duration and high levels of disease activity. Multiple studies have shown significant positive results in disease modulation, as well as limited side effects.

## Introduction and background

Thyroid eye disease (TED) is a devastating complication that can result in proptosis, vision loss, and facial deformation, all of which can be distressing to patients. TED occurs in phases starting with inflammation, leading to proptosis, which eventually switches to fibrotic stages that allow the disease to stabilize. Rundle’s curve shows the phases of the disease in relation to time, which predicts an approximately 18-month course of active disease before becoming static [1]. The traditional treatment for these patients is steroids or mycophenolate sodium [2] to help to reduce inflammation and then surgical correction once the disease has stabilized [3]. Strides have been made in recent years with the introduction of biological medications that can undergo immune modulation with limited side effects [4]. Tepezza® (teprotumumab-trbw) is a monoclonal antibody that targets the insulin-like growth factor-1 receptor (IGF-1R) and is upregulated on orbital fibroblasts and decreases activation in TED patients [1]. Tepezza® has been shown to have little to no adverse effects, in contrast to the multitude of adverse effects found in patients taking glucocorticoids for extended periods of time.

## Review

Thyroid eye disease

TED is related to activation of orbital fibroblasts and adipocytes and the production of glycosaminoglycans such as hyaluronic acid and chondroitin sulfate. This activation occurs due to upregulation of thyroid-stimulating hormone receptors (TSHRs) and IGF-1R, which get activated by auto-antibodies produced in Grave’s disease (GD) [5]. This activation leads to the expression of downstream genes and cytokines, which recruits inflammatory cells into the orbital tissue. These cells cause fibroblast stimulation and the secretion of glycosaminoglycans, resulting in adipose and muscle expansion and inflammation [3]. B and T lymphocytes have also been implicated in the pathophysiology of TED. Memory B cells have been shown to produce these antibodies and also activate T cells through antigen presentation; the stimulated T cells then produce cytokines within orbital fat and muscles. The active phase of TED has a predominant Th1 response, whereas the late phase is dominated by Th2 cells [1], which results in signs and symptoms such as retraction of the eyelids, swelling, proptosis, and diplopia [6].

Besides Tepezza®, multiple drugs have been researched for the medical management of TED, such as corticosteroids, rituximab, tocilizumab, mycophenolate, azathioprine, ciclosporin, and teprotumumab. Corticosteroids are primarily used to treat the active phase of the disease and have been shown to decrease proptosis by as much as 2 mm. Although high doses of Tepezza® were found to lead to significantly greater improvement in ocular motility, they were also associated with serious adverse effects, such as fulminant liver failure, cardiovascular complications, stroke, and death [7]. Studies on rituximab, a chimeric monoclonal antibody against CD20 on B cells, found that it did not have significant outcomes, and although it may play a role in severe disease refractory to corticosteroids, it should not replace them as an initial treatment [7]. Tocilizumab, a monoclonal antibody that binds to the interleukin-6 (IL-6) receptor, was found to have a slight disease-modifying effect by reducing orbital inflammation in patients with severe cases that are resistant to steroids [7]. Mycophenolate affects purine synthesis through the inhibition of inosine monophosphate dehydrogenase, thus causing immunosuppression. Although there have been mixed results from studies, it is currently thought that mycophenolate is efficacious when added to steroid treatment regimens that have shown only a partial response [7]. Although azathioprine, a purine analog that interrupts ribonucleotide synthesis, could possibly be of benefit due to its antiproliferative effects, additional studies are needed to determine its efficacy in TED [7]. Ciclosporin inhibits the transcription of interleukin-2 (IL-2), thus inhibiting T cell activation. Although corticosteroids were found to be superior treatment options, they may also be efficacious as a dual therapy when needed [7]. Tepezza® has shown promising results in the disease modification of TED and has been the first immune modulator approved by the United States Food and Drug Administration (FDA) for its treatment [7]. 

Pharmacokinetics/pharmacodynamics of Tepezza®

In TED patients, IGF-1R is upregulated in the fibroblasts of orbital tissue, allowing for selective targeting of the receptor to reduce TED inflammation and symptoms. Tepezza® specifically targets IGF-1R [3], and its relevant pharmacokinetics and pharmacodynamics have been studied in both healthy volunteers and TED patients. Pharmacokinetic properties refer to how the drug is absorbed, distributed, metabolized, and eliminated by the body. Tepezza® is administered intravenously over one hour every three weeks for a total of eight infusions [8]. Tepezza® exhibits dose-proportional pharmacokinetics, with the maximum concentration and area under the curve increasing proportionally with the dose [9]. The half-life of Tepezza® is approximately 14 days, which supports the three-week dosing interval, with the steady-state concentration achieved after the fifth dose [8]. After intravenous infusion of a single dose, the peak concentration is reached at the end of the infusion, with a mean volume of distribution of 6.9 L (range=6.4-9.9 L) [8]. The clearance of Tepezza® is approximately 0.3 L/day. Its bioavailability is unknown, as it is administered intravenously. Tepezza® was not found to be metabolized by the liver or kidneys, and its clearance is mainly through the reticuloendothelial system [10]. The pharmacokinetic profile of Tepezza® does not seem to be affected by age, gender, race, body weight, or concomitant medications [11]. However, as patients with moderate to severe renal impairment have increased exposure to Tepezza®, dosage adjustment is recommended in these patients [11]. The safety profile of Tepezza® is generally well tolerated, but up to 85% of patients experience side effects. The most common adverse events are muscle spasms (30%), alopecia (20%), diarrhea (10%), and hearing impairment (10%). In one study, 8% of patients had serious adverse events (i.e., seven teprotumumab-trbw recipients) [11]. Of these seven adverse events, three were deemed to be either treatment-related or possible treatment-related adverse events; these were severe diarrhea, an infusion-related reaction, and Hashimoto’s encephalopathy [11].

Tepezza® binds to IGF-1R, leading to the inhibition of fibroblast proliferation and collagen production. The downregulation of IGF-1R signaling also reduces the expression of proinflammatory cytokines such as IL-6 and tumor necrosis factor-alpha [12]. The inhibition of these cytokines reduces the infiltration of immune cells into the orbital tissue, leading to the attenuation of inflammation and tissue remodeling. The pharmacodynamics of Tepezza® are dose-dependent, with a maximal effect observed at a dose of 10 mg/kg [13]. Tepezza® is indicated for the treatment of active TED in adult patients. Active TED is defined as inflammation and tissue expansion in the eye orbit that is causing functional impairment or disfigurement [14]. Tepezza® is administered as an intravenous infusion over a period of one hour at a dose of 10 mg/kg body weight, once every three weeks for a total of eight infusions. The treatment duration is approximately six months [13].

Currently, there is an ongoing phase 3b/4 post-marketing clinical trial to monitor the safety and efficacy of Tepezza®, and also to document the tolerability of three different (three cohorts) medication dosing levels [14]. Cohort 1 will receive four infusions of teprotumumab (10 mg/kg for the first infusion and 20 mg/kg for the remaining three infusions) followed by four infusions of either a placebo (if there is a response to treatment) or teprotumumab 20 mg/kg (if there is no response). Cohort 2 will receive eight infusions (10 mg/kg for the first infusion and 20 mg/kg for the remaining seven infusions), and Cohort 3 will receive 16 infusions (10 mg/kg for the first infusion and 20 mg/kg for the remaining 15 infusions). The study is set to be finished in August 2025 and will provide great insight into the long-term safety and appropriate dosing(s) of Tepezza®. 

Tepezza® use for TED

As TED is a condition that affects the eyes of people with autoimmune thyroid disorders and can result in inflammation and swelling in muscles, tissues, and fat surrounding the eyes, it can result in symptoms such as eye pain, redness, swelling, double vision, and, in severe cases, vision loss. Tepezza® is the first FDA-approved drug specifically designed to treat TED, with the primary outcome of reducing proptosis [13]. The mechanism of action for Tepezza® involves direct targeting of IGF-1R, a protein that is over-expressed on the surface of cells in the orbit (eye socket) of TED patients. IGF-1R is involved in regulating cell growth, survival, and differentiation and is a key mediator of the inflammatory and fibrotic processes that drive TED [12]. Tepezza® works by binding to IGF-1R and blocking its activation by IGF-1, a hormone that promotes cell growth and division. By inhibiting IGF-1R signaling, Tepezza® reduces inflammation and fibrosis in the orbit, which leads to a reduction in the severity of TED symptoms. Although the exact mechanism by which Tepezza® achieves this effect is not fully understood, it is believed to involve several factors, as described below [15].

First, Tepezza® is thought to inhibit the proliferation and activation of fibroblasts, the cells that produce excess collagen and other extracellular matrix proteins that contribute to the fibrotic scarring in the orbit. This inhibition reduces the stiffness and resistance of the orbital tissues, allowing them to move more freely and relieving pressure on the eye [16].

Second, Tepezza® is believed to modulate the activity of immune cells such as T cells and B cells, which are involved in the autoimmune response that causes TED. By reducing the production of inflammatory cytokines and promoting the production of anti-inflammatory cytokines, Tepezza® helps to shift the balance toward a more tolerogenic (i.e., less inflammatory) environment in the orbit [17].

Third, Tepezza® may also have a direct effect on the muscles that control eye movement by reducing the infiltration of immune cells and fibroblasts into the muscle tissue and promoting muscle regeneration [16]. Tepezza® is administered intravenously over a period of several weeks, with each infusion lasting approximately one hour. The recommended dosage is 10 mg/kg body weight, with a total of eight infusions given over a 21-week period [13]. Clinical trials have shown that Tepezza® can significantly improve the signs and symptoms of TED, including proptosis (bulging eyes), diplopia (double vision), and eye pain, compared to a placebo [15].

Previous studies and findings

Several previous studies have shown the efficacy and safety of Tepezza® in the treatment of TED. The most notable areas of symptom improvement have been seen in the relief of proptosis, subjective diplopia, and soft-tissue inflammation (measured via Clinical Activity Score). Although the above-mentioned outcomes are the most common, the drug can also lead to reductions in extraocular muscle and orbital fat volume buildup secondary to the diagnosis of TED, with one study showing a significant reduction in extraocular muscle volume in six patients after taking the drug for 24 weeks [16]. Some may even expect a complete inactivation of the disease, with one pooled data analysis reporting numbers needed to treat (NNT) scores of 1.6 for proptosis response, 2.5 for diplopia response, 1.7 for overall response, and 2.5 for disease inactivation [18]. Altogether, the outcomes of Tepezza® administration have been shown to greatly enhance the quality of life of those living with TED.

Clinical efficacy

Of all the beneficial outcomes of the drug, the most significant response has been elicited in the improvement of proptosis, with one study citing an NNT as low as 1.36 for a positive proptosis response [16]. By definition, a proptosis response is a ≥2 mm reduction in the more severely affected eye while sparing the other. Another study showed that treatment with Tepezza® led to a higher mean best corrected visual acuity, a proptosis reduction of 4.7 mm, a 5.25-point improvement in Clinical Activity Score (CAS), and a 0.7-point improvement in Gorman diplopia score [12]. Echography results have also shown an extraocular muscle diameter reduction of 14.6% in patients treated with teprotumumab [19]. Another study reported 33% and 29% average decreases in orbital fat volume and extraocular muscle, respectively [20]. Another study showed that most patients, after completing treatment with Tepezza®, had upwards of 3- or 4-mm differences from baseline. According to the above-mentioned results, the efficacy of Tepezza® is comparable to that of orbital decompression surgery [21]. Patients diagnosed with dysthyroid optic neuropathy refractory to methylprednisolone showed improvement after treatment with Tepezza® [16,22]. The outcomes are also long-lasting, with another study showing that 33 out of 37 individuals initially treated with a placebo had an improvement in their proptosis when treated with Tepezza®, with 90.6% maintaining these results at a 48-week follow-up [23]. The drug is also effective in a diverse patient population, with similar results seen regardless of the individual’s sex or smoking status, and despite the duration of the disease [23].

Improvement in inflammatory response has also been recorded in CAS assessments of patients treated with Tepezza®. The CAS is a means to assess signs of acute inflammation through the presence of spontaneous orbital pain, gaze-evoked orbital pain, eyelid swelling that is considered to be due to active orbitopathy, eyelid erythema, conjunctival redness considered to be due to active orbitopathy, chemosis, and inflammation of the caruncle or plica. Each criterion present counts as a point in scoring, which ranges from 1 to 7. In one study, a CAS reduction of >3 points was more frequent in individuals treated with Tepezza® versus those treated with a placebo [23].

Subjective diplopia was also reported to improve with Tepezza® treatment in both chronic and active TED. One study showed that patients living with chronic TED experienced an improvement in symptomatic diplopia, with 68% reporting an increase in Gorman diplopia score of more than one point from the baseline [23]. Another study showed that 67% of patients experienced a clinically significant improvement in diplopia, with 47% having complete resolution following Tepezza® therapy [9]. A third study reported that diplopia response was seen in 68% of patients living with active TED following treatment with Tepezza®, as compared to 29% of those treated with a placebo [20]. Therefore, Tepezza® has been shown to be an effective drug for the treatment of TED, as summarized in Table 1.

**Table 1:** Clinical efficacy of Tepezza® TED: Thyroid eye disease

Author (Year)	Groups Studied and Intervention	Results and Findings	Conclusions
Douglas (2020) [16]	A 2019 multi-center, double-masked, placebo-controlled phase III clinical trial that investigated the effects of Tepezza^®^ on clinical outcome.	69% of patients receiving Tepezza^®^ vs. 20% receiving a placebo showed a reduced proptosis response at 24 weeks. Treatment onset was rapid, with 43% of patients receiving Tepezza^®^ vs. 4% of patients receiving the placebo reaching the primary outcome at 6 weeks of a reduced proptosis response.	These data suggest that Tepezza^®^ is useful in treating active moderate to severe TED.
Diniz (2021) [21]	A 2021 case series of 21 patients with clinical diagnosis of TED that investigated the effects of Tepezza^®^ on clinical outcome.	A reduction in proptosis of ≥2 mm was achieved in 71.4% of the sample, with some even showing improvement after refractory treatment of methylprednisolone therapy.	These data suggest that treatment of TED with Tepezza^®^ is associated with improvement in proptosis, extraocular motility, and CAS, and can be used as a replacement for current first-line therapy.
Sears (2021) [20]	A 2021 case series that investigated the post-interventional effects of Tepezza^®^ in patients with dysthyroid optic neuropathy.	Tepezza^®^ treatment showed rapid improvement of symptoms of dysthyroid optic neuropathy following two infusions.	These data suggest that Tepezza^®^ can be used in treating dysthyroid optic neuropathy refractory to high-dose corticosteroids, orbital radiation, and surgical decompression.
Ugradar (2022) [24]	A 2021 retrospective study that investigated the effects of infusions of Tepezza^®^ in chronic stable TED.	The mean reduction in proptosis for each study orbit was 3.5 mm and 3 mm for the fellow orbit. The CAS response was 90% for the study orbit and 87% for the fellow orbit. Of the 15 patients who had diplopia at baseline, 67% had a clinically significant response, whereas 47% had complete resolution following treatment.	These data suggest that Tepezza^®^ significantly reduces proptosis, inflammation, diplopia, strabismus and orbital soft tissue volume in patients with chronic stable TED.

Safety

In addition to being efficacious, Tepezza® is also safe, with few adverse events reported. The reported adverse events that do exist are usually mild to moderate in severity [16]. A pooled data analysis of patients undergoing treatment with Tepezza® found the most common antagonistic effects to be muscle spasms, hearing loss, and hyperglycemia. Some participants discontinued treatment in response to events such as diarrhea, infusion reaction, and Hashimoto’s encephalopathy [25]. Of note, some women taking the drug noticed menstrual changes such as amenorrhea, metrorrhagia, and dysmenorrhea. These findings were seen in up to 23% of menstruating women-six times higher than that seen in the placebo treatment group. However, these changes were mild and did not lead to any participants discontinuing therapy [26]. An additional study reported that the majority of patients experienced hearing loss, with less than half of those reporting subjective hearing loss noting a full resolution of symptoms. Another study reported four adverse events out of 37 patients in an initial course of treatment, with two events occurring after re-treatment [21]. Despite the above-mentioned events seen with Tepezza® treatment, it is important to note that far more adverse reactions are seen in the current first-line treatments of corticosteroid therapy or orbital decompression surgery [24]. Although orbital decompression surgery is avoided during active flare-ups of the disease, it is still notable that adverse reactions are much more common with the surgical approach.

Discussion

The results obtained with teprotumumab in the management of TED, as evidenced in this technical report, have been positive and given hope in the management of the disease. In previous reports, the general management of TED was well illustrated, and we and the authors were in agreement [4]. Key elements in the development of TED therapy were the recent acquisitions in its pathophysiology [20]. Arguably, the most important research developments have been the possibility that IGF-1R plays critical roles in the development of TED, the role of X in restoring immune tolerance in GD, and the use of improved research methodologies, with the refinement of mouse models of GD and TED [27]. The rationale for IGF-1R inhibition has become increasingly clear [28]. Studies on the pathophysiology of GD, and especially on the role of immune pathogenesis, have provided important support for the development of new therapeutic pathways [29].

It is important to acknowledge the basic information derived from basic research, with mouse models having a crucial role [30]. Mouse model studies have made possible novel treatments for GD and TED. For example, such studies confirmed that prophylactic administration of TSHR, a subunit protein, in genetically susceptible individuals could induce immune tolerance and provide protection for the future development of GD [31]. Studies on animals, combined with data on humans, have suggested that gut microbiota may play an essential role in the pathogenesis of GD and TED [32]. Therefore, even if teprotumumab represents an important step forward in treating GD and TED, there are many other aspects to explore. For example, refined animal models may provide excellent support for clinical activities and developments toward better disease management in these patients.

## Conclusions

Teprotumumab, commercialized as Tepezza®, is a monoclonal antibody medication that works by targeting the IGF-1R protein on cells in the orbit, reducing inflammation and fibrosis and improving the symptoms of TED. Its mechanism of action involves inhibiting fibroblast proliferation, modulating immune cell activity, and potentially promoting muscle regeneration. Although various immune modulators have been studied, Tepezza® is the first to be approved by the FDA for the treatment of TED. Compared to previous treatments for TED and their various adverse effects, Tepezza® is safe and effective in clinical trials and represents a significant advance in treatment.
